# The Influence of Vitamin C on Stromal and Epithelial Cells of the Pancreas in Malignant and Benign/Inflammatory Pancreatic Diseases: Protocol for a Scoping Review

**DOI:** 10.2196/91522

**Published:** 2026-06-22

**Authors:** Hanna Plischke, Yoshiaki Sunami, Artur Rebelo, Jörg Kleeff

**Affiliations:** 1 Department of Visceral, Vascular and Endocrine Surgery, University Hospital of Halle (Saale), Martin Luther University Halle-Wittenberg, Ernst-Grube-Straße 40, Halle (Saale), Saxony-Anhalt, 06120, Germany, +49 3455575290

**Keywords:** ascorbic acid, pancreatic neoplasms, pancreatitis, pancreatic stellate cells, tumor microenvironment

## Abstract

**Background:**

With a 5-year survival rate of less than 10% in most regions worldwide, pancreatic ductal adenocarcinoma (PDAC) has been considered as one of the deadliest tumor diseases. In addition, the incidence of acute and chronic pancreatitis is also increasing worldwide. Both diseases—tumorous and inflammatory—involve pronounced stromal changes, including stellate cell activation, fibrosis, immune cell recruitment, and a proinflammatory microenvironment that is able to drive malignant progression. Vitamin C is increasingly coming into focus as a treatment option, as it has the potential to act both as an antioxidant and anti-inflammatory agent on pancreatitis tissue and as a cytotoxic and modulatory agent on tumor-relevant signaling pathways in PDAC. While the influence of vitamin C on tumor cells has been extensively studied and classified, there is a lack of consideration in the context of stromal and epithelial cells in PDAC and benign/inflammatory pancreatic diseases.

**Objective:**

This review aims to map the currently scattered literature and, thus, detect possible gaps in research. The research question posed for this purpose is as follows: what data are available on the influence of vitamin C on pancreatic stromal cells and pancreatic cells in benign/inflammatory and malignant diseases of the pancreas?

**Methods:**

The scoping review will be conducted using the PRISMA-ScR (Preferred Reporting Items for Systematic Reviews and Meta-Analyses extension for Scoping Reviews) guidelines and the recommendations of the Joanna Briggs Institute. The main systematic search will use PubMed, MEDLINE, and Web of Science, with gray literature identified via the Bielefeld Academic Search Engine. Two independent reviewers will then conduct title and abstract screening, followed by full-text screening. The main inclusion criteria are (1) pancreatic cells in malignant and inflammatory pancreatic diseases, (2) vitamin C intervention, and (3) effects studied at the cellular level. Relevant data will be extracted using a standardized Microsoft Excel spreadsheet. This information will include the title, author, year, study design, model, cell lines, vitamin C isoform, concentration, treatment duration, control, outcomes for pancreatic and stromal cells, methods, and key findings. This list may be expanded during screening. Data analysis will be descriptive, complemented by thematic grouping and visual representations. In accordance with the PRISMA-ScR recommendations, a formal risk-of-bias assessment will not be performed.

**Results:**

As of April 13, 2026, a total of 475 database hits have been evaluated during the title and abstract screening process. The entire review process will extend from January 2026 to December 2026. Final results are expected in January 2027.

**Conclusions:**

This review aims to provide a comprehensive overview of the current state of knowledge in this field of research to structure it and, thus, highlight possible gaps in knowledge. This could serve as a basis for preclinical studies addressing specific gaps identified in this review to further elucidate the potential therapeutic role of vitamin C.

## Introduction

### Background

Pancreatic ductal adenocarcinoma (PDAC) has been a particular focus of basic research for many years, not least because of its consistently poor prognosis. The 5-year survival rate is below 10% in most regions worldwide [1]. Even though these figures have increased significantly over the last few decades, the prognosis remains very poor, especially for older patients with advanced stages of the disease, who make up most cases [2].

Since 1990, the absolute number of new cases of acute and chronic pancreatitis has also been steadily increasing worldwide. Despite improvements in diagnosis and treatment, which have resulted in at least a slight decline in age-adjusted incidence from approximately 38 to 33 per 100,000 inhabitants (1990 to 2021) [3], mortality remains significantly elevated for up to 10 years after an episode of pancreatitis compared to the general population [4]. The pancreatic stroma is a key topic in preclinical studies of pancreatitis. Recent data show that both acute and chronic pancreatitis lead to massive remodeling of the stroma, which includes activation of pancreatic stellate cells, pronounced fibrosis, recruitment of immune cells, and the formation of a proinflammatory microenvironment. Furthermore, it has been shown that remodeling of the pancreatic stroma creates an environment that can promote the development of pancreatic tumors [5,6]. This has led to considerable interest in the influence of the stroma on the pathogenesis of pancreatic diseases.

The tumor microenvironment (TME) is also an essential component in malignant processes as it serves as a host for all malignant and nonmalignant cells that could influence the development, progression, and therapeutic response of the tumor. Thus, the diversity of the TME is formed by tumor cells, fibroblasts, adipocytes, lymphocytes, vessels, dendritic cells, specifically cancer-associated fibroblasts (CAFs), and extracellular matrix, which influence each other and, through their cooperation, determine the biology of the tumor [7,8]. Over the past few years, there has been growing interest in vitamin C and its potential role as a therapeutic adjuvant not only in preclinical research. As the TME is characterized by oxidative stress, hypoxia, and metabolic dysregulation, redox-active molecules such as vitamin C are increasingly coming into focus as potential treatment options because they can directly influence these microenvironmental conditions. In the field of TME, vitamin C has also been shown to have immunomodulatory, epigenetic modifying, and cytotoxic functions [8].

Vitamin C is an organic compound that cannot be synthesized endogenously in humans due to the loss of function of L-gulonolactone oxidase. Its functions at physiological concentrations include the reduction of reactive oxygen species (ROS), participation in cell division processes, and the inhibition of aging processes [9]. It has been shown that vitamin C above physiological concentrations of 0.1 mmol/L [10] selectively attacks and kills cancer cells through a pro-oxidative effect, which functions, among other things, via the formation of ROS [11]. This and much higher concentrations can be easily achieved through intravenous injection of pharmacological ascorbate [12]. Functionally, extracellularly oxidized dehydroascorbate is taken up via glucose transporters (eg, glucose transporter 1) and reduced intracellularly by reduced glutathione back to ascorbic acid, whereby glutathione disulfide is subsequently reduced again by nicotinamide adenine dinucleotide phosphate. The resulting deficiency of reduced glutathione and nicotinamide adenine dinucleotide phosphate deprives tumor cells of their ability to neutralize ROS [13] and results in DNA damage and excessive strain on DNA repair systems [14]. Furthermore, it has been shown that ascorbate prevents hypoxia-inducible factor 1-alpha–dependent gene expression by acting on the active site of hypoxia-inducible factor 1-alpha hydroxylase as an additional mechanism of action [15]. Thus, it counteracts the tumor-typical induced HIF1A stabilization, which usually represents a survival and development advantage for the tumor during malignant progression [16]. Many studies report dose-dependent, tumor-specific cytotoxic effects in relation to PDAC [17-19].

In the case of pancreatitis, the antioxidant effects of vitamin C have recently become a focus of interest. It has been shown that patients with acute pancreatitis develop a vitamin C deficiency in the first 5 days, which correlates with the course of the disease in a dose-dependent manner [20]. However, the therapeutic effect is controversial. Many studies have shown a decrease in markers associated with oxidative stress, such as malondialdehyde, and a shorter length of hospital stay, but no benefit has yet been demonstrated in terms of organ failure or survival rates [21,22]. In addition to its function as an ROS scavenger, vitamin C also has a regenerative effect on other redox systems, activating, for example, the nuclear factor erythroid 2–related factor 2/NQO1/HO-1 signaling pathway, which can also reduce damage to pancreatic tissue by upregulating oxidative stress–inducible genes [21]. Furthermore, it has been shown that vitamin C in therapeutic concentrations can reduce elevated proinflammatory cytokines such as tumor necrosis factor-α and interleukin-6 [23].

The effects of vitamin C in relation to PDAC have already been discussed, summarized, and published in a multifaceted and structured manner at the tumor cell level without the clarified aim of reviewing data on CAFs or additional relevant TME components [12,24,25]. However, there is still no systematic overview of the relationships between stromal and epithelial cells of the pancreas when vitamin C is administered. In addition, in the field of benign and inflammatory pancreatic diseases, which are closely related to tumor development, the literature on models and concentrations of vitamin C is considered very diverse and has so far been comprehensively examined only in very few systematic literature studies [20]. Therefore, this scoping review aims to map the scattered evidence on this topic in a structured manner and identify possible research gaps and connections between various pancreatic diseases.

### Objectives

The question posed in a scoping review should generally be open and exploratory as the purpose of this form of literature review is to gain an overview of the existing literature on a topic. To construct the main question of this scoping review, the population, exposure, and outcome scheme was applied [26]. For the first part of this framework, both the epithelial cells of the pancreas and the surrounding pancreatic stroma were selected as the population in benign/inflammatory and malignant diseases of the pancreas. The selected intervention was vitamin C in physiological and therapeutic doses. Finally, any described influences on the populations included under “population” were considered outcomes.

Therefore, the primary research question resulting from the applied scheme was as follows: which data are available on the influence of vitamin C on pancreatic stromal cells and pancreatic cells in benign/inflammatory and malignant diseases of the pancreas?

To specify this broad topic and capture more precise outcomes, five subordinate questions were identified:

1. What cellular mechanisms are described in pancreatic cells in benign/inflammatory and malignant diseases of the pancreas as a result of vitamin C (effects on viability, apoptosis or necrosis, ROS levels, and hypoxia)?
2. What effects of vitamin C have been described on which components of the pancreatic stroma in benign/inflammatory and malignant diseases of the pancreas (effects on viability, apoptosis or necrosis, ROS levels, hypoxia, CAF activation, collagen synthesis, and immune cell activity)?
3. What experimental models have been used to investigate the effect of vitamin C on the populations described?
4. What concentrations and application forms have been used in the studies?
5. Which aspects of the interaction between vitamin C and pancreatic stromal cells or pancreatic cells have been investigated in the existing literature, and which areas have not yet been sufficiently researched?

## Methods

### Design

This review protocol is reported according to the PRISMA-P (Preferred Reporting Items for Systematic Reviews and Meta-Analyses Protocols) guidelines [27]. The final scoping review will be conducted based on the recommendations of the PRISMA-ScR (Preferred Reporting Items for Systematic Reviews and Meta-Analyses extension for Scoping Reviews), which are consistent with those of the Joanna Briggs Institute [28].

### Search Strategy

The search strategy is reported based on the PRISMA (Preferred Reporting Items for Systematic Reviews and Meta-Analyses) statement for reporting literature searches in systematic reviews, which is in part also applicable for scoping reviews [29]. In general, the search strategy should be both comprehensive and in depth to gain a broad overview of the research field but also to integrate all relevant items. This is also necessary to enable the existing data to be divided into specific categories during the review process. We will not impose any restrictions regarding the year of publication of potential papers to avoid bias in this regard. Therefore, the time span of the included publications refers to database inception up to the month of publication of the review.

First, an initial exploratory search was conducted in PubMed to identify relevant keywords and MeSH (Medical Subject Headings) terms and obtain an overview of the possible number of articles to be included. The terms obtained were then compiled into a search strategy according to the population, intervention, comparator, and outcome scheme, and the main systematic search was conducted in the PubMed, MEDLINE, and Web of Science Core Collection databases. Together, these databases provide a broad representation of the relevant literature. While additional databases may further expand coverage, the selected strategy is expected to capture most of the relevant literature. However, the search was supplemented by screening the reference lists of detected literature reviews and an additional broad search conducted in the Bielefeld Academic Search Engine to identify and include gray literature (Table 1). Rerunning of the database searches will be performed at least every 2 months to avoid missing recently published literature.

**Table 1.** Database-specific search strategies.

Database	Search string (example)	Limits or filters applied	Date of last search performed
PubMed and MEDLINE	(“Ascorbic Acid”[Mesh] OR “Vitamin C”[tiab] OR ascorbate[tiab] OR “L-ascorbic acid”[tiab] OR “Dehydroascorbic acid”[tiab] OR “ascorbic acid”[tiab]) AND (“Pancreatic Neoplasms”[Mesh] OR “Pancreatitis”[Mesh] OR pancreatitis[tiab] OR “acute pancreatitis”[tiab] OR “chronic pancreatitis”[tiab] OR “pancreatic cancer”[tiab] OR PDAC[tiab] OR “pancreatic ductal adenocarcinoma”[tiab] OR “pancreas injury”[tiab] OR “pancreas disease*”[tiab])	Language: English and German; species: human	April 13, 2026
Web of Science	TS=((“Vitamin C” OR “Ascorbic Acid” OR Ascorbate OR “L-ascorbic acid” OR “Dehydroascorbic acid”) AND (“Pancreatitis” OR “acute pancreatitis” OR “chronic pancreatitis” OR “Pancreatic Neoplasm*” OR “Pancreatic Cancer” OR “Pancreatic Ductal Adenocarcinoma” OR PDAC OR “pancreas injur*” OR “pancreas disease*”))	Language: English and German	April 13, 2026
Bielefeld Academic Search Engine	(“Vitamin C” OR “Ascorbic Acid”) AND (“Pancreatic Cancer” OR PDAC OR “Acute Pancreatitis” OR “Chronic Pancreatitis”)	Language: English and German	April 13, 2026

### Selection Process and Eligibility Criteria

After searching the databases, the identified hits will be exported to a suitable literature management program, which in this case will be EndNote (version 2025.2; Clarivate Analytics), and duplicates will be automatically and manually removed. This will be followed by a 2-stage screening process carried out by 2 independent reviewers, whereby a title and abstract review will first be conducted, followed by full-text screening. If the 2 reviewers disagree on the inclusion or exclusion of a hit and this disagreement cannot be resolved through discussion, it will be handled by a third experienced reviewer.

The inclusion criteria are based on the population, exposure, and outcome scheme created for our main research question. Accordingly, studies will be included whose population consists of pancreatic cells in malignant and inflammatory pancreatic diseases. Furthermore, vitamin C must be the subject of the intervention, and the effects of this intervention must have been examined at the cellular level.

The criteria that will lead to the exclusion of an article from the review process are outlined in Table 2.

**Table 2.** Exclusion criteria and rationale.

Exclusion criterion	Explanation
Editorials, commentaries, opinion pieces, and literature reviews	Only articles containing primary data will be included in this review.
Articles not available in full text and articles not written in English or German	Full-text availability and language comprehension are necessary for reliable data extraction.
Studies not investigating pancreatic stromal or epithelial cells	Studies focusing on other organs, tissues, or cell types do not correspond to this review’s population.
Studies that do not use human pancreatic cells or tissue or relevant experimental nonhuman models (eg, mice)	These outcomes are irrelevant for this review.
Studies in which vitamin C is investigated exclusively as a risk factor and is not used as an experimental or therapeutic intervention	The aim of this review is to determine the effects of vitamin C as an active intervention, not its association with disease risks or outcomes.
Studies in which vitamin C interventions cannot be distinguished from other interventions	Possible interference from other interventions disrupts the consolidation and interpretation of data.

**Figure 1.**
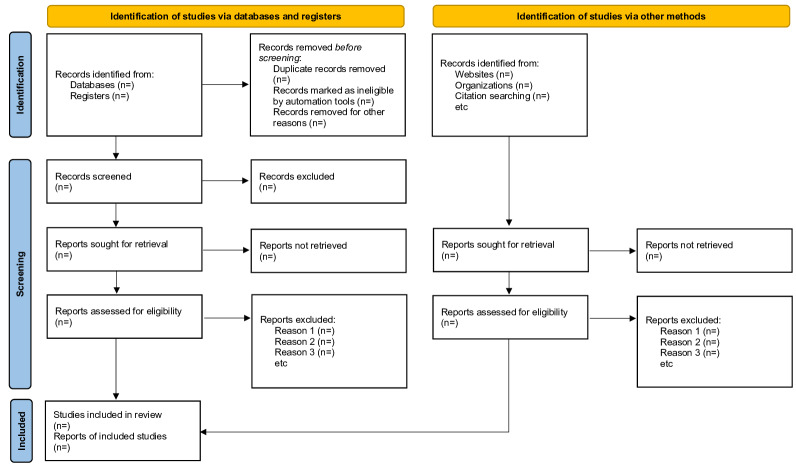
PRISMA (Preferred Reporting Items for Systematic Reviews and Meta-Analyses) 2020 flow diagram.

After excluding irrelevant or incomplete studies, the remaining articles will be included in the analysis. The entire selection process will be documented in a flow diagram in accordance with the PRISMA guidelines (Figure 1).

### Data Extraction

Extracted data from the included articles will be recorded using a prestandardized Microsoft Excel spreadsheet (version 16.104.1) and then finally evaluated for relevance based on the specified inclusion and exclusion criteria. If necessary, the elements queried in this spreadsheet can be expanded during the screening process.

However, the following elements will be extracted first from the included papers: title, author, year of publication, country, pancreatic disease, study design, model, cell lines used, vitamin C isoform, treatment periods with vitamin C, concentrations of vitamin C used, control, primary outcomes regarding pancreatic cells, primary outcomes regarding stromal cells, methods, and key findings.

### Data Synthesis and Quality Assessment

Data synthesis will be based on a predefined system of categories derived directly from the research questions. After evaluating the extracted data mentioned above, the included studies will be classified into the following categories: (1) disease (eg, PDAC, acute pancreatitis, and chronic pancreatitis), (2) cell type studied (eg, tumor cells, pancreatic stellate cells, and immune cells), (3) experimental model (eg, 2D cell culture, 3D model, and animal model), (4) type of vitamin C intervention (duration and concentrations), and (5) end points studied (eg, cell viability, apoptosis, ROS levels, and inflammatory markers).

**Figure 2.**
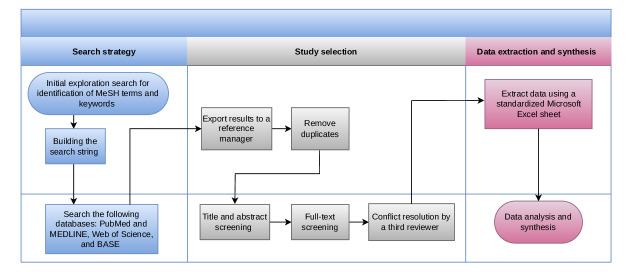
Illustration of the review process. BASE: Bielefeld Academic Search Engine; MeSH: Medical Subject Headings; WOS: Web of Science.

The composition of these categories is variable and can be expanded or reduced depending on the scope of the extracted data. Thus, each study can be assigned to a combination of these categories. On this basis, the analysis will be conducted through frequency analyses (eg, number of studies per disease, cell type, or end point), as well as through the identification of recurring combinations of categories. The results will then be presented in the form of tables and graphical representations. Prism (version 10.6.1; GraphPad Software) will be used for this purpose. This will allow us to visualize which combinations of categories have been frequently or rarely investigated in the literature, thereby enabling us to specifically identify research gaps (Figure 2).

Thematic groups will be formed by summarizing studies with comparable end points and intervention parameters. These groups will also be guided by the research questions established. Chapters based on research question 1 might look as follows: (1) cellular mechanisms influenced by vitamin C in PDAC and (2) cellular mechanisms influenced by vitamin C in inflammatory pancreatic diseases. Consequently, a summary of existing findings can be compiled within the corresponding thematic groups to present the current state of research in this field.

Neither a formal risk-of-bias assessment nor a formal assessment of the strength of the body of evidence will be performed as this is not necessary in accordance with the PRISMA-ScR recommendations [25].

## Results

A systematic search of the databases has already begun. As of April 13, 2026, a total of 475 database hits have been evaluated during the title and abstract screening process based on the specified inclusion and exclusion criteria. Data collection began in January 2026. To ensure that recently published literature is not missed, the databases are searched every 2 months. At the same time, the review of titles and abstracts, as well as the full-text screening, is currently being carried out. This process will continue until December 2026. Therefore, the results of this scoping review are expected in January 2027.

## Discussion

This scoping review is expected to show that current evidence on vitamin C in pancreatic diseases is predominantly focused on tumor cells, whereas stromal and inflammatory components remain underexplored. Therefore, our work could serve as an incentive to expand research in this area. By systematically analyzing and mapping the existing literature, including its coverage of all individual pancreatic compartments as well as different pancreatic diseases, this review extends beyond prior work. For example, it may be possible to determine whether there is sufficient preclinical evidence regarding the relationship between the TME and tumor cells during treatment with vitamin C. Similarly, controversies in preclinical findings could be identified and classified. Furthermore, it is of interest to highlight the frequency of methodologies used to uncover potential deficiencies in the effects of lower vs higher dosages or short-term vs long-term effects.

The inclusion of multiple databases, including gray literature, increases the likelihood of capturing the entirety of the available evidence. In addition to that, embedding the variety of research models, such as monocultures, coculture and 3D models, and animal models, in this review enables the recording and comparison of vitamin C–related mechanisms of action across these research methods. All processes in this literature review will be carried out strictly in accordance with the specifications and guidelines of the PRISMA-ScR statement [28]. This will result in a high level of methodological transparency and traceability.

Although the inclusion of a wide variety of models, vitamin C isoforms, concentrations, and time points is advantageous for interpreting the outcomes of this review, the resulting heterogeneity could limit the comparability of the data. This review will not perform a risk assessment, which means that methodologically stronger articles could be placed on equal footing with weaker articles. A limitation that occurs systematically in the field of TME research is the insufficiently homogeneous definition of the TME. Due to many different, interacting compartments that develop, are added, or are removed as knowledge progresses, there is no general consensus on the exact composition of the components of the tumor environment that are relevant for research [30]. This makes categorization significantly more difficult.

As this review maps a research field that has not been systematically investigated at that depth to date, it will make an important contribution to the current state of knowledge. These insights may serve as a base for future basic, preclinical, and clinical studies focusing on stromal remodeling, TME interactions, and dose-dependent effects of vitamin C, thereby supporting a more targeted evaluation of its therapeutic potential.

## Supplementary material

10.2196/91522 Checklist 1 PRISMA-S checklist.

## Abbreviations:

CAF: cancer-associated fibroblast
MeSH: Medical Subject Headings
PDAC: pancreatic ductal adenocarcinoma
PRISMA: Preferred Reporting Items for Systematic Reviews and Meta-Analyses
PRISMA-P: Preferred Reporting Items for Systematic Reviews and Meta-Analyses Protocols
PRISMA-ScR: Preferred Reporting Items for Systematic Reviews and Meta-Analyses extension for Scoping Reviews
ROS: reactive oxygen species
TME: tumor microenvironment
